# A Contralateral Eye Study Comparing Corneal Biomechanics in Subjects with Bilateral Keratoconus with Unilateral Vogt’s Striae

**Published:** 2017

**Authors:** Farshad ASKARIZADEH, Mohamad-Reza SEDAGHAT, Hadi OSTADI-MOGHADDAM, Foroozan NAROOIE-NOORI, Tahereh RAKHSHANDADI, Sattar RAJABI

**Affiliations:** 1 Refractive Errors Research Center, Mashhad University of Medical Sciences, Mashhad, Iran; 2 Department of Optometry, School of Paramedical Science, Mashhad University of Medical Sciences, Mashhad, Iran; 3 Cornea Research Center, Khatam‐Al‐Anbia Hospital, Mashhad University of Medical Sciences, Mashhad, Iran; 4 Department of Optometry, school of rehabilitation sciences, Zahedan University of Medical Sciences, Zahedan, Iran

**Keywords:** Corneal Biomechanics, Keratoconus, Vogt’s Striae, Corneal Biomechanics

## Abstract

The aim of this study was to analyze and compare corneal biomechanics in patients with bilateral keratoconus (KCN) with unilateral Vogt’s striae. In this prospective contralateral study, visual acuity, refraction, and corneal biomechanical parameters were evaluated in patients with bilateral KCN with unilateral Vogt’s striae using the Ocular Response Analyzer (ORA) (Reichert Inc., Buffalo, NY) and Corvis ST (Oculus Optikgeräte GmbH, Wetzlar, Germany). All patients underwent a comprehensive ophthalmic examination, including uncorrected distance visual acuity (UDVA), corrected distance visual acuity (CDVA), refraction (calculated by vectorial analysis), slit-lamp biomicroscopy, and Scheimpflug-based tomography. The patients enrolled in this study had a reliable diagnosis of bilateral clinical KCN with unilateral Vogt’s striae based on slit-lamp signs as well as corneal topographic/tomographic maps. Fifty patients aged 18 to 40 years were included in this study. There was a significant difference in all clinical (distance visual acuity and refraction) and corneal biomechanical parameters between KCN eyes with and without unilateral Vogt’s striae (all P < 0.05). However, there were no significant differences in peak distance (P = 0.291), corneal compensated intraocular pressure (IOPCC) (P = 0.08), and J45 (P = 0.131) between the two groups. Most corneal biomechanical parameters, except for peak distance, IOPCC, and J45, showed a significant difference between KCN eyes with and without unilateral Vogt’s striae. Vogt’s striae may cause corneal biomechanical deterioration. This information could be used in clinical practice.

## INTRODUCTION

Keratoconus (KCN) is a corneal ectatic, non-inflammatory, developmental, and progressive disorder [1, 2]. The prevalence of KCN ranges widely—from 50 to 2,300 cases per 100,000 persons—depending on the definition of KCN, geographic location, patient selection, diagnostic tool, and detection criteria [3, 4]. Although KCN typically starts unilaterally, the fellow eye eventually also becomes affected [5]. KCN is characterized by many clinical and subclinical manifestations that have a considerable impact on vision-related quality of life [1, 3-8]. On clinical examination using slit-lamp biomicroscopy, corneal Vogt’s striae have been considered one of the typical [7] and classical signs of KCN [9]. Vogt’s striae are usually parallel to the steep axis of the KCN cone, and present as fine vertical lines in the stroma [2, 9, 10]. They are also known as stress lines [9, 11], visible in moderate to advanced stages of KCN using high-magnification biomicroscopy [1, 2]. The Collaborative Longitudinal Evaluation of KCN (CLEK) study reported that 34% of patients with KCN have unilateral Vogt’s striae that are bilateral in 30% of the cases [12]. Some studies have evaluated the corneal characteristics related to Vogt’s striae [9, 13, 14]. Hollingsworth and Efron characterized the appearance of stromal banding patterns in patients with KCN using in vivo confocal microscopy (IVCM). They suggested that the alternating dark and light bands they observed represented Vogt’s striae. They also reported a positive correlation between the direction and location of the banding patterns in the deep stroma and the axis of the steepest keratometry measurements of the cornea using corneal topography [9]. In a study using IVCM and computerized videokeratography, Mocan et al. concluded that Vogt’s striae may be associated with corneal topographic and microstructural changes [13]. In another study, it was found that the viscoelastic nature of the cornea depends on the corneal extracellular matrix and stromal collagen fibrils [14]. The significance of corneal biomechanics in different ocular conditions is the main focus of many studies [15]. On the other hand, abnormal alterations of the collagen fibrils and interfibrillary material in the corneal stroma of patients with KCN may lead to biomechanical alterations in the cornea [16]. Considering the association of Vogt’s striae with other subclinical alterations in the cornea [13], questions have arisen about a possible association of Vogt’s striae with biomechanical corneal alterations in clinical KCN. There are two devices for in vivo assessment of corneal biomechanics. The first is the Ocular Response Analyzer (ORA) (Reichert Inc., Buffalo, NY), a dynamic bidirectional applanation device that can measure corneal biomechanical properties in terms of corneal hysteresis (CH) and corneal resistance factor (CRF) [17]. The other instrument is the Corvis ST (Oculus Optikgeräte GmbH, Wetzlar, Germany), which records the reaction of the cornea to a collimated air pulse with a newly developed high-speed Scheimpflug camera. This instrument records corneal deformation parameters for analyzing corneal biomechanics [17, 18]. In addition to deformation outcomes, Corvis ST shows the central corneal thickness (CCT) in the main printout. In vivo intraocular pressure (IOP) can be measured with both instruments. Since no previous studies have assessed corneal biomechanics in the Vogt’s striae, the aim of this contralateral eye study was to compare corneal biomechanical properties measured with the ORA and Corvis ST in patients with bilateral KCN with unilateral Vogt’s striae.

## MATERIALS AND METHODS

Fifty consecutive patients with bilateral KCN with unilateral Vogt’s striae were enrolled in this prospective contralateral eye study conducted from February 2017 to June 2017 at Sedaghat Eye Clinic in Mashhad, northeast of Iran. All cases were residents of Mashhad and were Iranian, with the same ethnicity. This study was approved by the Institutional Review Board/Ethics Committee of Mashhad University of Medical Sciences (registration number: 950806) and was conducted according to the tenets of the Declaration of Helsinki. All patients received information about the study and gave their written informed consent. All patients underwent a comprehensive ophthalmic examination, including full patient history, uncorrected distance visual acuity (UDVA), corrected distance visual acuity (CDVA), manifest and cycloplegic refraction (Topcon KR-1; Topcon Medical Systems, Inc., Tokyo, Japan), regularity status of the retinoscopic reflex, non-contact computerized tonometry (Topcon CT-1/CT-1P; Topcon Medical Systems, Inc.), ophthalmoscopy, slit-lamp biomicroscopy, and Scheimpflug-based tomography (Pentacam HR; Oculus Optikgeräte GmbH). The inclusion criterion was a reliable diagnosis of bilateral KCN with unilateral Vogt’s striae confirmed by an experienced corneal refractive surgeon (M.R.S.) based on slit-lamp signs, as well as corneal topographic/tomographic maps and an irregular retinoscopic reflex. Slit-lamp biomicroscopy at 40 magnification was used to identify true unilateral Vogt’s striae. The exclusion criteria were age below 18 and over 40 years old, previous eye surgery, corneal scarring, corneal vascularization, corneal inflammation, corneal opacity, history of herpetic keratitis, severe dry eye, glaucoma or glaucoma suspect, treatment with intraocular pressure-lowering drugs, and underlying autoimmune or systemic diseases. It should be mentioned that individuals with a history of corneal cross-linking or patients who wore contact lenses for less than 4 weeks before the beginning of the study were not included in the study group. Moreover, women who were in menstrual period and pregnant and nursing mothers were also excluded from the study. The eyes diagnosed as KCN suspect or forme fruste KCN were excluded from the study, too.

After selecting the study group, corneal biomechanics were evaluated in vivo using the ORA and Corvis ST. All corneal biomechanical measurements were done consistently based on the manufacturers’ instructions. The manufacturers’ representatives routinely check the calibration of the devices every 6 months. The ORA has the ability to measure CH, CRF, corneal compensated IOP (IOP_CC_), and Goldman correlated IOP (IOP_G_). In addition, the difference between CH and CRF (CH-CRF) as additional biomechanical descriptor [19, 20] was calculated for each subject. The mechanism of the ORA has been already described in other studies [15, 19, 20] and the repeatability and reproducibility of the ORA have been reported as acceptable [21-23]. As for the results of the ORA, the system monitors the entire process and produces a specific waveform. The ORA results consist of three consecutive measurements. If the measurements are of high quality according to the waveform score, only the reading with a better quality is included in the analysis. Ten minutes after ORA measurements, the Scheimpflug-based Corvis ST was used to measure corneal deformation outcomes. This device allows non-invasive imaging of the cornea’s dynamic deformation response to a puff of air using an ultra-high speed Scheimpflug camera [24-26]. The mechanism and repeatability of the Corvis ST in measuring corneal biomechanics have been presented elsewhere [25, 27-29]. All measurements were taken between 16:00 and 18:00 by one experienced optometrist (F.A). Three effective results were obtained at 5-minute intervals using the Corvis ST and the average of these values was used for analyses. Power vector analysis was performed to compare refraction between two study groups. The results of spherocylindrical refraction were converted to vectors expressed by three dioptric powers: 𝑀, 𝐽0, and 𝐽45, where 𝑀 was equal to the spherical equivalent of the given refractive error, and 𝐽0 and 𝐽45 were the two Jackson’s cross cylinder equivalents to the conventional cylinder. Cycloplegic refraction was recorded in the conventional manner (sphere, cylinder, and axis) and then this notation was converted to the coordinates of power vector as described by Thibos and Horner [30].

Statistical analyses performed using SPSS software version 22.0 (Chicago, IL, USA). The Kolmogorov–Smirnov test was used to determine the normality of the data. Paired-sample t-test used to compare the parameters with a normal distribution and the Wilcoxon signed rank test was used to compare non-parametric parameters between eyes with and without Vogt’s striae. Pearson correlation coefficients and Spearman’s rank correlation analysis tests used for correlative analyses. For all evaluations, a P-value less than 0.05 considered statistically significant.

## RESULTS

Our study was conducted on 50 patients with KCN [28 males (56%) and 22 (44%) females] to compare ocular and biomechanical parameters between eyes with and without Vogt’s striae. The mean age of the participants was 27.54 ± 6.78 years (range: 18 to 42 years). We considered KCN eyes with Vogt’s striae as group 1 and those without Vogt’s striae as group 2. As shown in Table 1, there were significant differences in UDVA, CDVA, maximum keratometry (Kmax), mean keratometry (Km) (all P < 0.001), and J0 (P = 0.001), but there was no significant difference in J45 (P = 0.131) between the two groups.

A comparison of the biomechanical parameters measured by the ORA between the two study groups is shown in Table 2. The KCN eyes with Vogt’s striae had lower CH, CRF, and IOPG (all P < 0.001) but greater CH-CRF (P = 0.001) than those without Vogt’s striae.

A comparison of the biomechanical parameters measured by the Corvis ST between the two study groups is shown in Table 3. The KCN eyes with Vogt’s striae had lower radius, CCT, and IOP (P < 0.001, P < 0.001, and P = 0.012, respectively) and greater DA (P < 0.001) than those without Vogt’s striae. Peak distance and deformation amplitude (DA) were higher in KCN eyes with Vogt’s striae than in those without Vogt’s striae (P = 0.291 and P < 0.001, respectively). 

The correlations between ORA-derived measurements and Corvis ST parameters for the two study groups are shown in Table 4.

**Table 1:** Contralateral Comparison of Basic Parameters between Keratoconus Eyes with Vogt’s Striae and those without Vogt’s Striae

Parameter	With Vogt’s striae		Without Vogt’s striae			
	**Mean ± SD**	**Range**	**Mean ± SD**	**Range**	**Mean difference**	**P-value**
Sph (D)	-3.26 ± 3.02	-10.00 to +1.75	-1.31 ± 2.07	-7.75 to +1.50	-1.95 ± 2.80	< 0.001*
Cyl (D)	-5.46 ± 2.23	-9.50 to -1.75	-2.42 ± 2.14	-8.50 to 0.00	-3.04 ± 2.44	< 0.001 *
SE (D)	-5.98 ± 3.55	-14.00 to -0.12	-2.44 ± 2.75	-11.37 to +0.88	-3.46 ± 3.26	< 0.001 *
J0 (D)	1.27 ± 2.26	-4.00 to +8.93	0.48 ± 1.05	-1.76 to +4.09	0.83 ± 1.90	0.001 *
J45 (D)	0.32 ± 1.83	-3.71 to +3.13	-0.19 ± 1.12	-3.25 to +2.62	0.55 ± 2.62	0.131 *
UDVA (LogMAR)	0.71 ± 0.47	0.1 to 1.60	0.43 ± 0.45	0.00 to 1.60	0.28 ± 0.43	< 0.001 *
CDVA (LogMAR)	0.33 ± 0.30	0.00 to 1.00	0.09 ± 0.11	0.00 to 0.40	0.25 ± 0.27	< 0.001 *
Km^ǂ^ (D)	50.00 ± 4.41	43.20 to 63.40	46.28 ± 2.99	40.00 to 53.60	3.72 ± 3.32	< 0.001^║^
Kmax^ǂ^ (D)	58.99 ± 5.65	48.10 to 71.00	52.00 ± 5.47	44.10 to 67.40	6.99 ± 4.58	<0.001^║^

Sph: sphere, Cyl: cylinder, SE: spherical equivalent, 𝐽0: Jackson’s cross cylinder, axes at 0 and 90 degrees, 𝐽45: Jackson’s cross cylinder, axes at 45 and 135 degrees, UDVA: uncorrected distance visual acuity, CDVA: corrected distance visual acuity, Km: mean keratometry, Kmax: maximum keratometry, D: diopter, LogMAR: logarithm of the minimum angle of resolution, SD: standard deviation,

There were no missing data. A P-value < 0.05 is statistically significant.

ǂ was measured by Pentacam,

* Wilcoxon signed-rank test,

║ paired-sample t-test, bold values are significant.

**Table 2:** Contralateral Comparison of Biomechanical Parameters Measured by the Ocular Response Analyzer between Keratoconus Eyes with Vogt’s Striae and those without Vogt’s Striae.

Parameter	With Vogt’s striae		Without Vogt’s striae			
	**Mean ± SD**	**Range**	**Mean ± SD**	**Range**	**Mean difference**	**P-value**
CH (mmHg)	8.31 ± 1.08	5.40 to 10.40	8.89 ± 1.24	6.10 to 11.60	-0.59 ± 1.05	< 0.001^║^
CRF (mmHg)	6.75 ± 1.38	4.30 to 10.10	7.72 ± 1.46	4.10 to 11.70	-0.97 ± 1.08	< 0.001 ^║^
CH-CRF (mmHg)	1.57 ± 0.69	-0.10 to 2.70	1.18 ± 0.65	-0.60 to 2.70	0.39 ± 0.72	0.001*
IOP_cc _(mmHg)	12.96 ± 1.79	11.00 to 17.40	13.58 ± 1.92	10.50 to 17.70	-0.63 ± 2.26	0.080 *
IOP_G _(mmHg)	10.79 ± 1.28	10.00 to 14.90	11.77 ± 1.36	11.00 to 18.00	-0.98 ± 1.36	< 0.001 *

CH: corneal hysteresis, CRF: corneal resistance factor, IOPCC: corneal compensated intraocular pressure, IOPG: Goldman correlated intraocular pressure, SD: standard deviation.

Bold values are significant. There were no missing data. A P-value < 0.05 is statistically significant.

* Wilcoxon signed-rank test,

║ paired-sample t-test.

**Table 3:** Comparison of Biomechanical Parameters Measured by Corvis ST between Keratoconus Eyes with Vogt’s Striae and those without Vogt’s Striae.

Parameter	With Vogt’s striae		Without Vogt’s striae			
	**Mean ± SD**	**Range**	**Mean ± SD**	**Range**	**Mean difference**	**P-value**
Peak distance (mm)	5.11 ± 0.23	4.67 to 5.73	5.14 ± 0.26	4.17 to 5.69	-0.03 ± 0.20	0.291
Radius (mm)	5.29 ± 0.84	3.10 to 6.65	6.40 ± 0.94	3.66 to 8.31	-1.11 ± 0.87	< 0.001
DA (mm)	1.18 ± 0.11	1.01 to 1.43	1.12 ± 0.11	0.88 to 1.36	0.06 ± 0.11	< 0.001
CCT (m)	457.42 ± 38.14	371.00 to 542.00	479.42 ± 36.17	397 to 550	-22.00 ± 22.06	< 0.001
IOP (mmHg)	13.76 ± 1.16	12.00 to 17.00	14.15 ± 1.28	11.00 to 17.50	-0.39 ± 1.05	0.012

DA: deformation amplitude, CCT: central corneal thickness, IOP: intraocular pressure, SD: standard deviation.

Bold values are significant. There were no missing data. A P-value <0.05 is statistically significant.

║ paired-sample t-test.

In KCN eyes with or without Vogt’s striae, CH had a negative correlation with DA (P = 0.004, r = -0.400 and P = 0.001, r = -0.455, respectively). In KCN eyes without Vogt’s striae, CH had a positive correlation with radius (P = 0.012, r = 0.354). In the KCN eyes with or without Vogt’s striae, CRF had a correlation with radius (P = 0.006, r = -0.385 and P = 0.001, r = 0.469, respectively) and DA (P < 0.001, r = -0.570 and P < 0.001, r = -0.630, respectively). According to Table 4, in KCN eyes with or without Vogt’s striae, CH-CRF had a negative correlation with radius (P = 0.004, r = -0.398 and P = 0.007, r = -0.376, respectively) and DA (P = 0.001, r = 0.462 and P < 0.001, r = 0.548, respectively). In KCN eyes without Vogt’s striae, CH-CRF was related to peak distance (P = 0.001, r = 0.358).

**Table 4:** Correlative Coefficients of the Ocular Response Analyzer and Corvis ST Parameters in KCN Eyes with Vogt’s Striae and those without Vogt’s Striae.

Parameter	Peak distance (mm)		Radius (mm)		DA (mm)	
	**P-value**	**r**	**P-value**	**r**	**P-value**	**r**
CH (mmHg)						
With Vogt’s striae	0.103*	-0.230	0.113 *	0.231	0.004 *	-0.400
Without Vogt’s striae	0.849 *	0.028	0.012 *	0.354	0.001 *	-0.455
CRF (mmHg)						
With Vogt’s striae	0.102 *	-0.240	0.006 *	0.385	< 0.001 *	-0.570
Without Vogt’s striae	0.203 *	-0.183	0.001 *	0.469	< 0.001 *	-0.630
CH-CRF (mmHg)						
With Vogt’s striae	0.428^ǂ^	0.115	0.004 ^ǂ^	-0.398	0.001 ^ǂ^	0.462
Without Vogt’s striae	0.001 *	0.358	0.007 *	-0.376	< 0.001 *	0.548

CH: corneal hysteresis, CRF: corneal resistance factor, DA: deformation amplitude.

Bold values are significant. There were no missing data. A P-value < 0.05 is statistically significant.

* Pearson’s correlation coefficient analysis,

ǂ Spearman’s rank correlation coefficient analysis.

## Discussion

The significance of corneal biomechanics in KCN has been reported in different studies [28, 31, 32]. However, to our knowledge, no study has investigated the impact of Vogt’s striae on the biomechanical properties of the cornea in KCN eyes. We designed this contralateral eye study to evaluate and compare corneal biomechanics in patients with bilateral KCN with or without Vogt’s striae measured with the ORA and Corvis ST. Our results showed significant differences in many biomechanical outcomes, except for peak distance, IOP_CC_, and J45, between bilateral KCN eyes with or without Vogt’s striae. The majority of biomechanical metrics in this study showed considerable weakening and deterioration in KCN patients with Vogt’s striae in comparison to KCN without Vogt’s striae. Moreover, visual acuity (corrected and uncorrected) and refractive components (spherical equivalents and J0) were different between patients with KCN with or without Vogt’s striae. In addition to ORA measurements, we calculated the CH-CRF as an additional biomechanical descriptor and the results showed a difference between KCN eyes with Vogt’s striae and those without Vogt’s striae. Mocan et al. reported an association between the presence of Vogt’s striae and other microstructural corneal alterations in KCN using IVCM. They found significant differences in refractive errors in spherical equivalents, astigmatic errors, and steepest corneal curvatures between patients with Vogt’s striae and those without Vogt’s striae [13]. Similar findings were obtained in our study. However, it should be noted that Mocan et al. evaluated refraction and corneal curvature but did not study corneal biomechanics, while we evaluated clinical and biomechanical parameters in a contralateral eye study. In another study, Hollingsworth and Efron investigated the correlation between the orientation of the bands (Vogt’s striae) in the stroma and the steepest keratometry axis of the cornea using corneal topography [9]. They did not compare the clinical and subclinical findings between patients with KCN with Vogt’s striae and those without Vogt’s striae.

We found a significant difference in IOP measured by the Corvis ST between KCN eyes with Vogt’s striae and those without Vogt’s striae. In addition, IOP_G_ measured by ORA, but not IOP_CC_, was significantly different between KCN eyes with Vogt’s striae and those without Vogt’s striae. According to Lanza et al. [33], the IOP results may be related to CCT and keratometry values between the two study groups. Indeed, the CCT and keratometry values between KCN eyes with Vogt’s striae and those without Vogt’s striae were different in our study. The results of the present study showed that UDVA and CDVA were worse in eyes with Vogt’s striae than in eyes without Vogt’s striae. Moreover, we found that K_m_ and K_max_ were higher in eyes with Vogt’s striae than in eyes without Vogt’s striae. CCT measurements showed a thinner cornea in eyes with Vogt’s striae. Additionally, the majority of findings in our study showed that the presence of Vogt’s striae in KCN eyes could make the cornea weaker as compared to KCN eyes without Vogt’s striae. According to the findings of this study, CH-CRF was higher in KCN eyes with Vogt’s striae than in eyes without Vogt’s striae. A review of the literature shows that CH-CRF has a higher value in KCN versus normal eyes. Moreover, the CRF is higher in non-keratoconic cornea than CH [19, 20]. Our study showed higher CH-CRF in KCN eyes with Vogt’s striae than in KCN eyes without Vogt’s striae. Based on reasoning strategies in clinical decision-making, it can be concluded that the higher the CH-CRF, the worse the corneal biomechanics. 

In the present study, we investigated the possible correlation between biomechanical metrics driven by the ORA and Corvis ST (Table 4). In KCN eyes with Vogt’s striae, there was a negative correlation between CH and DA, CRF, and DA as well as between CH-CRF and radius, and a positive correlation between CRF and radius as well as between CH-CRF and DA. On the other hand, in KCN eyes without Vogt’s striae, there was a positive correlation between CH and radius, CRF and radius, CH-CRF and DA as well as CH-CRF and peak distance, and a negative correlation between CH and DA, CRF and DA as well as CH-CRF and radius. Our data appears to suggest that in clinical practice, we should consider the presence or absence of Vogt’s striae in the corneal stroma when evaluating the corneal biomechanics in KCN. The differences and correlations between biomechanical parameters in KCN eyes with or without Vogt’s striae can lead us to better understanding of the corneal biomechanics of KCN eyes. We can consider, in general, worse biomechanical parameters for KCN eyes with Vogt’s striae than in eyes without Vogt’s striae.

One important strength of our study is the comprehensive evaluation of corneal biomechanics in KCN eyes with or without Vogt’s striae. However, one limitation is that we did not use IVCM. In summary, the corneal biomechanical parameters measured by the ORA and Corvis ST showed significant differences between KCN eyes with Vogt’s striae and those without Vogt’s striae. Even though the mechanism for corneal biomechanics measurements in the ORA and Corvis ST is different, the results of corneal biomechanics were different between the two study groups. The findings of the present study can be used in clinical evaluation, monitoring, and treatment of patients with KCN with or without Vogt’s striae. 
